# Pregabalin as a Pain Therapeutic: Beyond Calcium Channels

**DOI:** 10.3389/fncel.2020.00083

**Published:** 2020-04-15

**Authors:** Sascha R. A. Alles, Stuart M. Cain, Terrance P. Snutch

**Affiliations:** ^1^Michael Smith Laboratories, University of British Columbia, Vancouver, BC, Canada; ^2^Djavad Mowafaghian Center for Brain Health, University of British Columbia, Vancouver, BC, Canada; ^3^Department of Anesthesiology and Critical Care Medicine, University of New Mexico School of Medicine, Albuquerque, NM, United States

**Keywords:** pregabalin (PGB), chronic pain, therapeutic targets, calcium channel, hyperexcitability

## Abstract

Initially developed to generate new treatments for epilepsy, gabapentin, and pregabalin (“gabapentinoids”) were engineered to mimic the action of GABA and to modulate GABA metabolism. Rather than their intended pharmacological action on GABA neurotransmission, instead, they exhibit a high affinity for the α2δ-1 and α2δ-2 subunits of voltage-activated calcium channels, wherein binding of gabapentinoids inhibits cellular calcium influx and attenuates neurotransmission. Despite a lack of activity on GABA levels, gabapentin and pregabalin are effective at suppressing seizures and subsequently approved as a new class of antiepileptic therapy for partial-onset epilepsy. Through the same hypothesized molecular mechanism and by controlling neuronal hyperexcitability, gabapentinoids demonstrate clear efficacy in pain management, which has arguably been their most extensively prescribed application to date. In this review, we focus on pregabalin as a second-generation gabapentinoid widely employed in the treatment of a variety of pain conditions. We also discuss the wider functional roles of α2δ subunits and the contributions that pregabalin might play in affecting physiological and pathophysiological processes.

## Pharmacology and Protein Interactions of Pregabalin

### Pregabalin Is a High-Affinity α2δ Subunit Ligand

The alpha-2-delta (α2δ) subunits were first described as auxiliary subunits of the high voltage-activated calcium channels and are encoded by four genes: α2δ1, α2δ-2, α2δ-3 and α2δ-4. α2δ-1-3 are expressed in neurons as well as heart and skeletal muscle tissue (Gong et al., 2001), whereas α2δ–4 is found in non-neuronal cell types such as the retina and endocrine tissues (Dolphin, 2013). Pregabalin selectively binds to the α2δ-1 and α2δ-2 subunits and despite its original design as a GABA mimetic, pregabalin does not affect GABA_A_ or GABA_B_ receptor activity (Li et al., 2011).

Interestingly, α2δ-1 expression appears to co-localize mainly with excitatory neurons whereas α2δ-2 is found largely in inhibitory neurons (Cole et al., 2005). Recently, it has been shown that gabapentin exerts differential effects on putative excitatory and inhibitory spinal dorsal horn neurons (Alles et al., 2017). It is thus possible that pregabalin has different effects on excitatory compared to inhibitory neurons through a mechanism involving α2δ-1 and α2δ-2 subunits. This distinction may represent an important pregabalin-mediated therapeutic action since excitation-inhibition imbalance occurs in the spinal cord and brain in chronic pain states such as diabetic neuropathy and chronic pain accompanying multiple sclerosis (Coull et al., 2005; Petrou et al., 2012; Kuner, 2015; Potter et al., 2016).

Most pain studies addressing the pregabalin mechanism of action have focussed on α2δ-1 binding. α2δ-1 itself has been implicated in neuropathic pain and it is upregulated in the dorsal root ganglia (DRG) and primary afferent nerve terminals following nerve injury in rodent models (Luo et al., 2001). Mice overexpressing α2δ-1 show behavioral hypersensitivity to pain (Nguyen et al., 2009) and further, pregabalin analgesic efficacy is lost in transgenic mice carrying a α2δ-1 R217A mutation in the gabapentinoid binding site (Field et al., 2006). The α2δ-1 R217A mutation results in a significant decrease in calcium current through Ca_v_2.2 (N-type) channels, possibly due to a change in structural interaction with the pore-forming α1 subunit; however, this mutation does not appear to alter α2δ-1 function *in vivo* (Field et al., 2006).

α2δ-1 expression is enriched in the spinal dorsal horn, periaqueductal gray (PAG), anterior cingulate cortex (ACC), insula and amygdala (Stahl et al., 2013). The ACC and PAG are strongly implicated in nociceptive (dorsal horn) and emotional aspects of pain processing (Peirs and Seal, 2016) and pregabalin efficacy as a pain drug is most likely in part due to its action on α2δ-1 in these areas. In support, pregabalin reduces the release of glutamate in the spinal dorsal horn in rodent pain models (Kumar et al., 2013) and also reduces activation of the insula and amygdala during emotional processing that plays a role in the experience of pain (Aupperle et al., 2011).

Distinct from high voltage-activated calcium channel interactions, it has been demonstrated that astrocyte-secreted factors called thrombospondins (TSPs) are endogenous ligands of α2δ-1. The TSP-α2δ-1 complex is thought to form a “synaptogenic signaling complex” (Eroglu et al., 2009). In terms of neuropathic pain pathogenesis, it has been hypothesized that when α2δ-1 is upregulated, there is increased excitatory synaptogenesis driving increased neuronal network excitability. This mechanism is supported by earlier studies demonstrating that “central sensitization” of pain circuitry, a form of pathological learning, underlies neuropathic pain (Woolf, 1983; Costigan et al., 2009).

**Figure 1:**
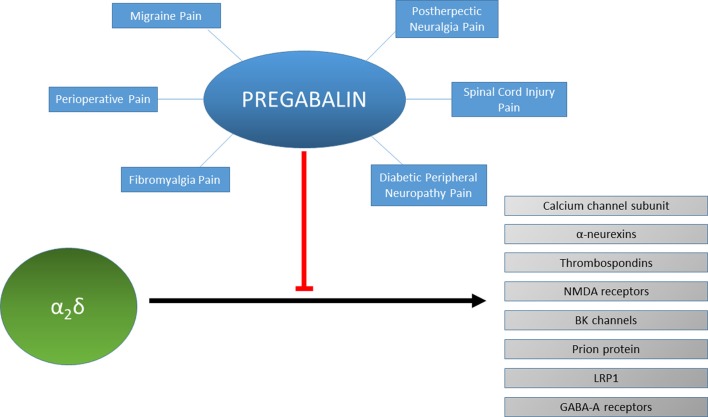
Protein interactions of α2δ and their inhibition by pregabalin. Pregabalin is used to treat different types of chronic pain such as migraine, spinal cord injury pain or fibromyalgia. The effectiveness of pregabalin for treating each type of chronic pain for each individual patient may be reflected by the modulation of the protein interactions of α2δ such as with neurexins, thrombospondins, NMDA receptors, BK channels, prion proteins, LRP1 or GABA-A receptors.

Several other recent findings have shed light on novel functions of α2δ-1 in the context of inflammatory and neuropathic pain (see Figure 1). For example, it has been shown that α2δ-1 interacts with the N terminus region of BK potassium channels (Zhang et al., 2018). Co-expression of BK channels with Cav2 channels reduces cell surface expression and whole-cell calcium current density due to competition for α2δ-1 for binding. Reproducing this situation *in vivo* results in analgesia in both inflammatory and neuropathic pain models. Pregabalin may enhance an interaction between endogenous BK and Cav2 channels through its α2δ-1-binding site. Alternatively, pregabalin might sequester free α2δ-1 and prevent it from associating with Cav2 channels before BK channels. Another study demonstrated that α2δ-1 also associates with NMDA receptors to form complexes and that the physical interaction promotes synaptic expression of α2δ-1-NMDA receptor complexes (Chen et al., 2018). Notably, gabapentin targets these complexes to alleviate neuropathic pain. Although pregabalin was not tested in this study, it would likely operate by a similar mechanism in this context. Given the broad functions and roles of the NMDA receptor, this mechanism might help to explain how the gabapentinoids successfully reduce cellular hyperexcitability in various systems and disease states (Paoletti et al., 2013).

It has been shown that synaptic cell-adhesion molecules α-neurexins regulate Ca^2+^ influx through Cav2.1 channels with α2δ-1 (Brockhaus et al., 2018). In cultured hippocampal neurons alpha-neurexins with α2δ-1, but not α2δ-3, facilitated calcium currents of Cav2.1. Therefore, in disorders involving perturbation of the Cav2.1 channel function, pregabalin may help correct α2δ-1-α-neurexin interactions.

Associations between α2δ and other proteins have been demonstrated including prion proteins (Alvarez-Laviada et al., 2014), GABA-A receptors (Geisler et al., 2019) and an important N-type calcium channel trafficking-related protein called low-density lipoprotein (LDL) receptor-related protein-1 (LRP1; Kadurin et al., 2017). LRP1 binding to α2δ-1 has been suggested to involve the binding pocket for gabapentin, therefore LRP-1 may occlude the binding site for pregabalin as well. With regards to a potential role of LRP-1 in pain pathophysiology, one study has shown that the perisciatic application of LRP-1 agonists facilitated an increase in nociceptive thresholds by local application of opioids (Yang et al., 2016). However, no studies of LRP-1 in chronic pain patients have been reported to date.

### Acute vs. Chronic Actions of Pregabalin: Comparing Clinical and Basic Science Findings

Clinically relevant concentrations of gabapentin have been calculated from plasma levels in neuropathic pain patients at approximately 15–30 mg/L (88–175 μM; Juenke et al., 2011). Generally, pregabalin displays higher potency than gabapentin due to a higher affinity for α2δ-1 (Field et al., 2006). In humans, the maximum recommended single oral dose of pregabalin is 300 mg, which reaches a maximum plasma concentration of roughly 40 μM within 1 h and a maximum CSF concentration of 2.6 μM within 8 h (Buvanendran et al., 2010). Steady-state levels of pregabalin after repeated administration of 600 mg daily vary between 17–51 μM in plasma (Bockbrader et al., 2010a) and are ~1%–30% lower in CSF (Bockbrader et al., 2010b).

In animals, pregabalin exerts acute actions on calcium channel-mediated currents recorded from neuromuscular junctions and Calyx of Held excitatory synapses (Uchitel et al., 2010). However, other studies have suggested that pregabalin actions are mainly chronic through reducing calcium channel trafficking from DRG to primary afferent terminals synapsing in the spinal dorsal horn (Bauer et al., 2009, 2010; Hendrich et al., 2012). It is not clear whether acute or chronic actions may predominate in a clinical setting since some patients only report benefit from pregabalin after several days (consistent with a chronic mode of action; Sharma et al., 2010), whereas some patients derive benefit with pregabalin when provided perioperatively (consistent with an acute mode of action; Schmidt et al., 2013). Further studies that attempt to correlate basic science with clinical findings are required to hone in on a consensus for the mechanism(s) of action of the gabapentinoids.

The general mechanism of pregabalin physiological cellular action involves a decrease in the release of several types of neurotransmitters including glutamate, noradrenaline, and substance P (Fink et al., 2000; Dooley et al., 2007; Brawek et al., 2008). In one study pregabalin inhibited K^+^-evoked acetylcholine, noradrenaline and serotonin release without affecting dopamine release in human neocortex (Brawek et al., 2008). There is little else to suggest that pregabalin is selective for a specific neurotransmitter type. Experiments on rodent neocortical slices have examined the effects of gabapentin on norepinephrine release evoked by a high K^+^ challenge in the presence of different voltage-gated calcium channel blockers (Dooley et al., 2002). This study showed that gabapentin appeared to show a preference for action on P/Q-type channels. Several studies other show that the gabapentinoids also reduce neurotransmitter release in the spinal dorsal horn wherein N-type channels are the most predominant high voltage-activated subtype (Coderre et al., 2005; Kumar et al., 2013; Alles et al., 2017). These differences likely speak to the complexity of both gabapentinoid actions and the distinct roles of specific calcium channel subtypes in controlling release, although are generally consistent with the notion that the main therapeutic action of these drugs involves reducing calcium-mediated neurotransmitter release to reduce neuronal excitability.

Conversely, Baba et al. (2016) demonstrated that clinically relevant concentrations of bath applied pregabalin have no acute inhibitory effects on evoked intracellular Ca^2+^ responses of dorsal horn neurons under naïve or neuropathic conditions. This is in contrast to previous studies showing that pregabalin can reduce the release of glutamate in the dorsal horn following systemic or intrathecal injection in both naïve and neuropathic conditions (Coderre et al., 2005; Kumar et al., 2013). These discrepancies may be due to differences in the method of delivery of pregabalin and/or in the assays employed for determining pregabalin effects. For example, calcium responses are an indirect indicator of neurotransmitter release and the stimulation paradigm for evoking calcium responses may not be physiologically relevant. Also, there are time course and concentration issues to consider when comparing bath applied with systemically injected pregabalin.

Functional MRI studies show that pregabalin decreases activation of the insula and amygdala during emotional processing (Aupperle et al., 2011). Paradoxically, in the same study pregabalin produced an increase in ACC activation, which might be predicted to exacerbate pain sensation. The authors argue that this could be a top-down effect on modulation of emotional processing.

The α2δ subunits in part contribute to setting presynaptic calcium channel abundance and release probability in central synapses (Hoppa et al., 2012). Pregabalin may exert its effects *via* a mechanism that reduces release probability, which would serve to dampen excitability in neuronal circuits. A recent article has demonstrated that gabapentin can promote regeneration following spinal cord injury *via* a blockade of α2δ-2, which inhibits axon growth (Sun et al., 2020). It has also been demonstrated that presynaptic α2δ-2 controls postsynaptic clustering of GABA_A_ receptors and that when this process is disrupted may cause excitation-inhibition imbalance as observed in chronic pain states and neuropsychiatric diseases (Geisler et al., 2019). As such, pregabalin therapeutic effects may in part be due to its effect of α2δ-2 subunits, serving to promote repair, aberrant axonal wiring, and shifts in excitation-inhibition imbalance.

There is more work to be done on improving understanding of the role of α2δ subunit subtypes in basic physiology and disease states (for a review on emerging neuronal functions and post-translational processing of α2δ subunits, see Geisler et al., 2015; Dolphin, 2018).

## On-Label Uses for Pain

Pregabalin is approved by the FDA for use to treat peripheral neuropathic pain, generalized anxiety disorder, and as an adjunct therapy for epilepsy (Dworkin and Kirkpatrick, 2005; Bagal et al., 2013). Although the gabapentinoids are considered first-line treatment options for neuropathic pain, the number needed to treat (NNT) for 50% pain relief in patients is 7.7 for pregabalin and 7.2 for gabapentin (Finnerup et al., 2015). In part, the high NNT likely reflects the complexity and many distinct types of chronic pain together with the high variability in pain phenotypes and severity across patients.

The most common causes of chronic pain are fibromyalgia, diabetic peripheral neuropathy, postherpetic neuralgia (nerve damage following shingles), and neuropathic pain associated with spinal cord injury (Dworkin and Kirkpatrick, 2005). The term neuropathic pain is applied to any acute or chronic pain syndrome thought to result from aberrant central or peripheral somatosensory pain processing (Portenoy and Cruciani, 2009). Common symptoms of neuropathic pain sensation include a sensation of burning or electric shock, hyperalgesia, hyperpathia, dysesthesia, allodynia, and paresthesia. Pregabalin is an effective treatment for neuropathic pain irrespective of the length of time the patient has displayed symptoms (Pérez et al., 2017). For this review we discuss the use of pregabalin for treating specific types of chronic pain, however we recognize that pain phenotypes exist trans-etiologically and that classifying patients according to disease is not necessarily predictive of drug response.

### Pregabalin in Fibromyalgia

Fibromyalgia is a form of idiopathic or “nociplastic” pain that causes widespread musculoskeletal pain for which no alternative cause can be identified, presenting as diffuse hyperalgesia and/or allodynia and is the most common cause of widespread body pain in humans (Derry et al., 2016; Sluka and Clauw, 2016). It is more prevalent in women and prevalence increases with age, with up to approximately 4% of women and 0.01% of men reported in the general population (Heidari et al., 2017). Fibromyalgia in a number of peripheral pain conditions is predicted to occur as a result of enhanced nociceptive signaling and the development of CNS-mediated symptoms related to sleep/fatigue, mood and memory deficits (Sluka and Clauw, 2016). Secondary fibromyalgia can also occur, when comorbid with a disease-causing constant nociceptor activation (Clauw, 2014).

Current therapeutic interventions for fibromyalgia include both non-pharmacological and pharmacological approaches, often integrated for best effect (Clauw, 2014). Drug therapies cover a wide range of pharmacological approaches and include opioids, cannabinoids, tricyclic antidepressants, selective serotonin reuptake inhibitors (SSRIs) and gabapentinoids (Fitzcharles et al., 2013). Pregabalin is recommended for fibromyalgia at a dose starting at 25 mg to 50 mg per day increasing to a maximum dose of 300 mg to 450 mg per day (Boomershine, 2010). A recent review examining clinical data from multiple studies found a significant reduction in moderate to severe pain from fibromyalgia at doses of 300 mg to 600 mg pregabalin compared to placebo (Derry et al., 2016). The authors also noted adverse reactions in approximately 10% of individuals treated with pregabalin compared to placebo.

Mechanistically, pregabalin is predicted to reduce fibromyalgia pain by preventing sensory propagation of nociception due to inhibition of calcium channels and neurotransmitter release in the ascending pain pathway (Biggs et al., 2014). Also, pregabalin has been shown to directly reduce glutamate and glutamine levels in the posterior insula in fibromyalgia patients, also interfering with its functional connectivity to the default mode network (DFN; Harris et al., 2013). This finding is of particular interest given that the DFN, which is a group of brain regions normally involved in self-referential thinking and autobiographical memory, shows increased activity in patients with fibromyalgia (Buckner et al., 2008; Napadow et al., 2010). It is uncertain whether enhanced basal activity in the DFN is a causative factor of neuropathy in fibromyalgia, or simply that increased sensory input increases basal activity in the DFN.

Studies of fibromyalgia patients using occipital nerve stimulation (ONS) show disturbed conditioned pain modulation (Ahmed et al., 2018). Pregabalin has been shown to exert effects on descending pain circuitry in nerve-injured mice (Bee and Dickenson, 2008), therefore these central effects of pregabalin may contribute to its effectiveness for treating fibromyalgia.

### Pregabalin in Diabetic Peripheral Neuropathy

Diabetic peripheral neuropathy is the most common complication in Type II diabetes mellitus, resulting from nerve damage caused by chronically high blood sugar levels (Çakici et al., 2016). Nerve damage is predicted to occur *via* microvascular injury of blood vessels supplying nerves. Symptoms usually appear first in the lower extremities (feet and legs) and can appear later in the upper extremities, manifesting as numbness, tingling, burning sensations, sharp pain and loss of balance (Çakici et al., 2016). Prevalence can occur at up to 29% in populations with Type II diabetes (Davies et al., 2006; Bansal et al., 2014) and is more common in non-Caucasians and women (Marmiroli and Cavaletti, 2016).

Similar to fibromyalgia, tricyclic antidepressants and opioids are commonly used to treat the symptoms of diabetic peripheral neuropathy (Javed et al., 2015; Tajti et al., 2016) and a capsaicin patch has been shown to display similar efficacy to oral drugs, albeit with limits in tolerability (van Nooten et al., 2017). Both gabapentin and pregabalin are also effective treatments, with pregabalin recommended at a dose of 300 mg to 600 mg daily resulting in a significant reduction in pain scores (Juhn et al., 2015).

Pregabalin suppresses the activity of excitatory primary afferent fibers that carry nociceptive information to the spinal dorsal horn (Biggs et al., 2014). However, at clinically relevant concentrations acute pregabalin does not affect calcium entry in dorsal horn A- and C- fibers induced by dorsal root ganglion stimulation in either normal rats or a rat model of streptozotocin-induced painful diabetic peripheral neuropathy (Baba et al., 2016). This is in contrast to gabapentin that has been shown to inhibit synaptic transmission from DRG to the dorsal spinal cord in the same pain model (Patel et al., 2000). However, it takes several days for pregabalin to induce anti-nociceptive effects in patients with diabetic peripheral neuropathy and, therefore it seems likely that a chronic mechanism of action involving the downregulation of pro-nociceptive proteins and/or synaptic plasticity may explain its effects (Freeman et al., 2008; Bauer et al., 2010).

### Postherpetic (Herpes Zoster) Neuralgia

The *varicellar zoster* virus causes chickenpox, which when reactivated from its latent state up to decades later results in herpes zoster, a condition manifesting as a painful rash, also known as shingles (Fashner and Bell, 2011). While the virus can be treated with acyclovir and acyclovir-generic antivirals postherpetic neuralgia is a painful sensation that can persist for months or even years following an attack of herpes zoster without a continuing rash (Mallick-Searle et al., 2016). A high proportion (>90%) of adults show serology indicative of prior herpes zoster infection (Kilgore et al., 2003) and up to 20% of those suffering an initial infection go on to develop postherpetic neuralgia (Klompas et al., 2011). Nerve fibers may be damaged due to the inflammatory response accompanying the reactivation of the varicellar zoster virus (Woolf and Mannion, 1999; Mallick-Searle et al., 2016). The resulting pain can manifest as constant or intermittent without a stimulus, or as hyperalgesia.

Like other neuropathic pain disorders, tricyclic antidepressants are commonly used to address pain transmission, or basic topical analgesics, such as lidocaine can be used to suppress nociception in postherpetic neuralgia. The gabapentinoids are also first choice drugs, with pregabalin displaying similar efficacy when postherpetic neuralgia patients were switched from gabapentin to pregabalin at one-sixth of the dose (Ifuku et al., 2011). Pregabalin is recommended at 150 mg to 600 mg daily and consistently improves pain scores in patients with postherpetic neuralgia (Frampton and Foster, 2005). Of note, pregabalin combined with topical lidocaine (5% patch), resulted in significant pain reduction in patients who previously did not respond to either drug (Nalamachu and Morley-Forster, 2012).

### Neuropathic Pain Associated With Spinal Cord Injury

Following an injury to the spinal cord, motor and sensory deficits occur, occasionally leading to paralysis. These often occur concurrently with nociceptive or neuropathic pain reported in approximately two-thirds of patients (Hagen and Rekand, 2015). The source of neuropathic pain can occur at, above or below the spinal cord injury, arising from damage to nerve roots, the site of spinal cord injury itself or pathophysiological alterations to neurons in the pain transduction pathway (Hagen and Rekand, 2015). Pregabalin (150–600 mg/day) is effective at treating patients with neuropathic pain associated with spinal cord injury, also improving sleep, anxiety and general patient status (Siddall et al., 2006; Cardenas et al., 2013). With regards to possible mechanism of action, it has recently been shown that gabapentinoids promote sprouting and regeneration of corticospinal axons in mice following spinal cord injury (Sun et al., 2020). Pregabalin also enhances myelin repair and attenuates glial activation in a demyelination model of rat optic chiasm, which may also contribute to its effectiveness in treating damage caused by spinal cord injury (Daneshdoust et al., 2017).

## Off-Label Uses of Pregabalin

Aside from neuropathic pain, pregabalin is frequently prescribed for off-label use at the discretion of the attending physician.

### Migraine Headache

Migraine is a debilitating painful condition described as a recurrent headache, often accompanied by visual disturbances and nausea (Burstein et al., 2015). The condition can occur accompanied by a genetic component but also spontaneously and the underlying mechanisms are still controversial (Ferrari et al., 2015). Spreading depolarization (SD) is a pathophysiological neurological phenomenon proposed to underlie migraine aura, yet it is not always observed in patients (Lauritzen et al., 2011). This may suggest that SD does not occur in all migraines, or perhaps that cortical electrodes are insufficient to detect SD-mediated voltage shifts that occur in isolation subcortically. SD is a multifactorial cascade, which can be prevented by blockade of synaptic transmission, but is also critically dependent on diffusion of extracellular neuroactive factors that are released by affected neurons during prolonged depolarization (Pietrobon and Moskowitz, 2014). As a result, the pharmacological prevention of SD is problematic without also severely affecting normal neurotransmission. Still, triptans and ergot derivatives act at serotonin receptors and are commonly used as abortive therapies in migraine *via* predicted vasoconstriction of blood vessels (Goadsby et al., 2002).

Interestingly, onabotulinumtoxin A is effective as a therapy for the treatment of chronic migraine, which is characterized by a headache frequency of 15 episodes per month or more (Frampton, 2012; Lipton et al., 2016). The mechanism of action of onabotulinumtoxin A is to inhibit excitatory neurotransmitter release by blocking synaptic vesicle fusion to the plasma membrane (Durham and Cady, 2011). Pregabalin also attenuates neurotransmitter release, albeit by a different mechanism than onabotulinumtoxin A, therefore there is a strong argument for its use as a potential chronic migraine treatment.

Although initial studies indicated that gabapentin may be effective in the treatment of migraines, it has since been shown to be only marginally effective (Mathew et al., 2001; Hoffmann et al., 2010; Linde et al., 2013; Zain et al., 2013; Mulleners et al., 2015). Conversely, pregabalin has shown encouraging early, clinical results in the treatment for migraine headaches (Calandre et al., 2010; Pizzolato et al., 2011; Bakhshandeh Bali et al., 2015) and in mouse models effectively suppresses SD velocity and propagation to subcortical brain structures following an acute dose (Cain et al., 2017). While promising findings, further clinical evidence is required to establish a definitive role for pregabalin in migraine therapy.

### Pre-operative Administration for Post-operative Pain

Human studies have demonstrated a clear anti-nociceptive effect of pregabalin when administered as an acute dose before surgery. A single dose (75–150 mg) has demonstrated significant efficacy in reducing post-operative pain in for example orthopedic surgery (Akhavanakbari et al., 2013; Dong et al., 2016), lumbar dissectomy (Spreng et al., 2011), septoplasty (Sagit et al., 2013), thyroidectomy (Bindu et al., 2015), and hysterectomy (Rajappa et al., 2016). These clinical findings provide strong support for the acute mechanism of pregabalin efficacy. However, variables such as the degree of surgery invasiveness, length of post-operative follow-ups and low statistical power often make these studies difficult to evaluate by meta-analysis.

Visceral pain is an important clinical problem such as that associated with irritable bowel syndrome (Camilleri and Boeckxstaens, 2017). Pregabalin has been shown to reduce visceral hypersensitivity in a mouse model of opioid-induced hypersensitivity (Bannister et al., 2011), therefore may prove an effective treatment for visceral pain.

## Future Perspectives

The gabapentinoids constitute a distinct pharmacological class of pain therapeutic with a well-defined molecular target yet a still poorly understood physiological mechanism of action. There remain many unanswered questions. For example, whether pregabalin acts as an allosteric modulator of TSPs *via* α2δ-1 or has a direct inhibitory effect on TSPs. Also to be better defined are the exact physiological and pathophysiological contributions of pregabalin binding to α2δ-2 subunits in central and peripheral pain pathways. Controversies also remain regarding the role of pregabalin as an acute vs. chronic therapeutic in terms of distinct or overlapping mechanisms correlating with clinical efficacy. Also, while a large body of data has defined the effects of pregabalin in DRGs and the spinal cord, the actions of pregabalin in the brain are less well understood.

The effectiveness of pregabalin in the clinic varies widely with some chronic pain patients deriving benefits more quickly than others and in response to much lower doses (Sharma et al., 2010). Studies that attempt to correlate these inter-patient differences with individual genetic, epigenetic and/or phenotypic characteristics are urgently required. This may lead to the development of more effective pain therapeutics as well as a deeper understanding of pain neurobiology.

Off-label uses for pregabalin are numerous while FDA approved uses for pregabalin remain limited to a few conditions raising the question of whether FDA approval will be sought for use in emerging disorders or whether off-label use will become increasingly common.

Newer gabapentinoids have been developed with mirogabalin undergoing clinical trials for neuropathic pain (Calandre et al., 2016). Whether the clinical efficacy of this new derivative will differ sufficiently from gabapentin and pregabalin remains to be determined.

## Author Contributions

All authors contributed to all aspects of the review including literature review and manuscript writing and revision.

## Conflict of Interest

The authors declare that the research was conducted in the absence of any commercial or financial relationships that could be construed as a potential conflict of interest.
